# Spatio-temporal variation in European starling reproductive success at multiple small spatial scales

**DOI:** 10.1002/ece3.1615

**Published:** 2015-07-22

**Authors:** Daisy Brickhill, Peter GH Evans, Jane M Reid

**Affiliations:** 1 Institute of Biological & Environmental Sciences, School of Biological Sciences, University of AberdeenZoology Building, Tillydrone Avenue, Aberdeen, AB24 2TZ, U.K; 2 School of Ocean Sciences, University of BangorMenai Bridge, Anglesey, Wales, LL59 5AB, U.K

**Keywords:** Demography, double brood, life-history variation, seasonal reproductive success, *Sturnus vulgaris*

## Abstract

Understanding population dynamics requires spatio-temporal variation in demography to be measured across appropriate spatial and temporal scales. However, the most appropriate spatial scale(s) may not be obvious, few datasets cover sufficient time periods, and key demographic rates are often incompletely measured. Consequently, it is often assumed that demography will be spatially homogeneous within populations that lack obvious subdivision. Here, we quantify small-scale spatial and temporal variation in a key demographic rate, reproductive success (RS), within an apparently contiguous population of European starlings. We used hierarchical cluster analysis to define spatial clusters of nest sites at multiple small spatial scales and long-term data to test the hypothesis that small-scale spatio-temporal variation in RS occurred. RS was measured as the number of chicks alive ca. 12 days posthatch either per first brood or per nest site per breeding season (thereby incorporating multiple breeding attempts). First brood RS varied substantially among spatial clusters and years. Furthermore, the pattern of spatial variation was stable across years; some nest clusters consistently produced more chicks than others. Total seasonal RS also varied substantially among spatial clusters and years. However, the magnitude of variation was much larger and the pattern of spatial variation was no longer temporally consistent. Furthermore, the estimated magnitude of spatial variation in RS was greater at smaller spatial scales. We thereby demonstrate substantial spatial, temporal, and spatio-temporal variation in RS occurring at very small spatial scales. We show that the estimated magnitude of this variation depended on spatial scale and that spatio-temporal variation would not have been detected if season-long RS had not been measured. Such small-scale spatio-temporal variation should be incorporated into empirical and theoretical treatments of population dynamics.

## Introduction

Quantifying the pattern and magnitude of spatial variation in demography within and among populations and subpopulations is key to understanding and predicting population dynamics (Pulliam 1988; Rodenhouse et al. 1997; Hanski 1998). A population's spatial nature can be considered to comprise two components: its physical structure (i.e., the spatial locations and arrangements of individuals) and, superimposed upon this, spatial variation in demographic rates (i.e., reproduction, survival, and movement). Both the physical and demographic components of spatial structure can fundamentally affect population dynamics. Physical structure can influence extinction risk within predator–prey (Chivers et al. 2014), host–parasite (Aparicio et al. 2004), and single-species systems (Hanski 1998). Spatial variation in demography can cause source-sink dynamics where sink areas with population growth rates (*λ*) of less than one are sustained by immigration from source areas where *λ* > 1 (Pulliam 1988; Pulliam and Danielson 1991). Population regulation can occur when spatial variation in demography is combined with preemptive occupancy of more productive sites (Rodenhouse et al. 1997; McPeek et al. 2001; Sergio and Newton 2003; Tschumi et al. 2014), and hence when physical and demographic structures interact. Given these potential effects, a first requirement for any study aiming to understand population demography and dynamics should be to quantify a population's spatial nature in terms of both physical structure and superimposed demographic variation (Pulliam 1988; Hanski 1998; Banda and Blanco 2009).

Spatial variation in demography should not be considered in isolation from temporal variation. This is because temporal variation can exacerbate or negate the impact of spatial variation on population structure and dynamics. For example, Johnson (2004), demonstrated source-sink dynamics within a population of neotropical rolled-leaf beetles (*Cephaloleia fenestrata*), but these dynamics were ephemeral and only occurred when flooding rendered certain areas sinks. The role of spatial variation and consequent source-sink dynamics in driving population change may therefore be over- or underestimated if insufficient time is considered. Furthermore, even minimal spatial variation in demographic rates could impact population dynamics if the pattern of spatial variation remains consistent over sufficient time. A population or spatially restricted subpopulation with *λ* that is fractionally but consistently under one, and insufficient immigration to compensate, will ultimately go extinct (Pulliam and Danielson 1991). Full assessment of the magnitude and potential consequences of spatial and temporal variation in demography, and the interactions between them, therefore requires demographic variation to be quantified across appropriate spatial and temporal scales.

Scale is a critical consideration in any such spatio-temporal analysis (Levin 1992; Chave 2013; Sutherland et al. 2013; Sandel 2014). In species with discrete breeding seasons, these provide a biologically appropriate temporal unit (Gaillard et al. 2000; Coulson et al. 2001). However, appropriate spatial scales are often less clear, and key processes can be inaccurately estimated or go undetected if biologically inappropriate spatial scales or divisions are chosen (Wiens 1989; Orians and Wittenberger 1991; Coulson et al. 1997). For example, Cowen et al. (2006) modeled connectivity between populations of reef fish and showed that ecologically relevant scales of larval dispersal were smaller than expected and hence that populations were more isolated than previously thought. In many cases, there may not be a single “correct” spatial scale because different mechanisms causing demographic variation may operate at different scales (Levin 1992; De Knegt et al. 2011). For example, demography may be influenced by local habitat at small scales but by predation at larger scales (De Roos et al. 1991). In the absence of clear a priori knowledge of appropriate spatial scale, the most insightful approach to understanding spatially explicit population dynamics may be to quantify demographic variation across multiple candidate scales and compare results (Sandel and Smith 2009; Yeager et al. 2011).

Defining appropriate spatial scale(s) has proved problematic (Talley 2007; Cornell and Donovan 2010; Sandel 2014). Many empirical studies aiming to quantify spatial variation in demography focus on populations that comprise distinct geographical or biological subunits, and hence where a priori spatial subdivisions appear obvious to observers (Saracco et al. 2010). For example, many studies consider archipelagos (e.g., Saether et al. 1999; Sonsthagen et al. 2012), territorial species (e.g., Nystrand et al. 2010), or distinct habitat types (e.g., Ozgul et al. 2006; Russell and Ruffino 2011). However, such analyses do not quantify demographic variation within these coarse subunits. Few studies have quantified small-scale spatial variation in demography within populations or areas that lack such obvious internal divisions. Exceptions include studies by Coulson et al. (1997, 1999) where, rather than imposing a priori divisions, cluster analysis of individual locations was used to define spatial substructuring in red deer (*Cervus elaphus*) and Soay sheep (*Ovis aries*). Cluster analysis on survival probability itself revealed small-scale spatial variation in survival in red-billed choughs (*Pyrrhocorax pyrrhocorax*, Reid et al. 2006). The paucity of such studies, coupled with theory suggesting that dynamics of spatially heterogeneous populations may differ substantially from more contiguous populations (Thomas and Kunin 1999), highlights the need to quantify spatial variation in demography within populations that are not, to human observers, obviously divided into discrete subunits.

Reproductive success (RS) is one key demographic rate that can cause substantial variation in *λ* (Saether and Bakke 2000). Furthermore, RS might be expected to show small-scale spatial variation, potentially reflecting numerous local environmental impacts such as food abundance or availability, microclimate, or topography. RS is often estimated by measuring the success of a single breeding attempt per season (Donovan et al. 1995; Arlt et al. 2008). However, many animals can potentially make multiple breeding attempts during a single reproductive season, and the number of attempts can greatly influence an individual's total RS and overall *λ* (Wilson and Arcese 2003; Cornulier et al. 2009; Sim et al. 2011). Despite the widespread potential for multibrooding, relatively few studies have quantified spatio-temporal variation in RS across entire breeding seasons rather than solely across single breeding attempts (Fortescue 1999; Husby et al. 2009).

**Figure 1:**
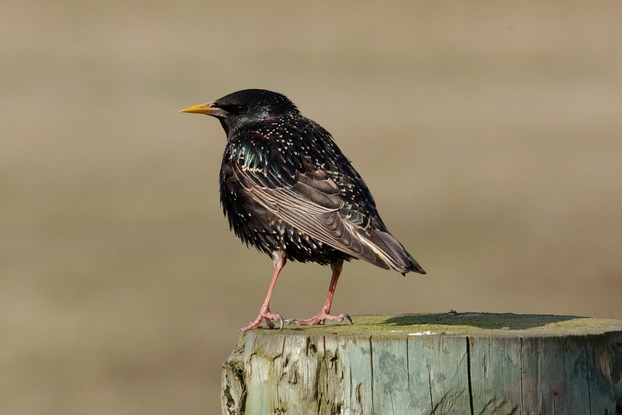
The European starling, *Sturnus vulgaris*. Photo: © Rebecca Nason

We used long-term data from a small and apparently contiguous population of European starlings (*Sturnus vulgaris*, Fig. 1) on Fair Isle, Scotland, to quantify very small-scale spatio-temporal variation in RS. We used hierarchical cluster analysis (HCA) to define spatial groupings (“clusters”) of nest sites at multiple spatial scales and thereby objectively describe the population's physical structure. We then quantified spatio-temporal variation in RS, defined as the numbers of offspring produced per first brood and over the entire breeding season, across clusters and years. We thereby test the overall hypotheses that reproductive success varies at a small spatial scale within an apparently contiguous population, and that conclusions regarding such variation depend on the choice of scale and the metric of reproductive success. Specifically, we answer four primary questions: (1) Does first brood RS vary at small spatial scales within the study population? (2) Is the pattern of spatial variation stable over time, such that certain clusters of nests have consistently higher RS than others across years? (3) Do these patterns of spatio-temporal variation remain the same when season-long rather than solely first brood RS is considered (i.e., if multiple breeding attempts are included)? (4) Does the spatial scale considered affect the estimated magnitude of spatial variation in RS?

## Materials and Methods

### Study system

Starlings are semi-colonial breeders and do not defend a breeding territory other than the immediate nest site (Feare 1984). They forage communally, chiefly on ground-living invertebrates in open grasslands with short vegetation (Feare 1984; Smith and Bruun 2002). Starlings can rear two broods per breeding season and the frequency of second broods varies geographically (Evans 1980; Feare 1984; Cramp et al. 1993).

**Figure 2:**
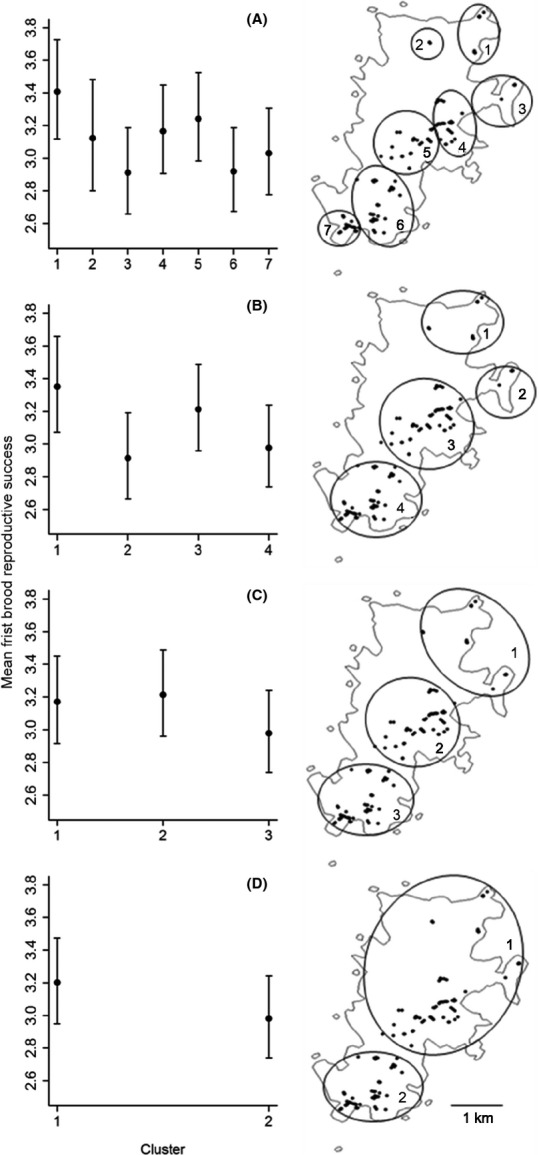
Locations of European starling nest sites across Fair Isle (black dots on maps). Clusters of nest sites (shown in circles) were created using hierarchical cluster analysis which defines spatial clusters based on linear distances among sites. The mean predicted first brood reproductive success (RS_FL_) ±1 SE is shown for each cluster. Clusters and mean RS_FL_ are shown for four spatial scales (panels A–D) with clusters numbered north to south at each scale. Y-axes are standardized to allow comparison across spatial scales. Sample size (i.e., total number of observations of RS per cluster across years) is shown above predicted means. The analysis was carried out using a 29-year dataset and predictions are shown for an average year.

A resident population of starlings inhabiting Fair Isle, Scotland (59°31′ 52.17″°N, 1°37′ 53.09″°W, ca.5 × 2.5 km, 750 ha), has been studied since 1980. Study nests are located in semi-natural cavities in stone walls and rock piles distributed across the island (Fig. 2). The availability of these landscape features on the island means that nest sites are unlikely to be limited. Vegetation comprises largely mixed rough grazed grassland and heather. Nests in the coastal cliffs are not included in the study area.

Every year from 1980 to 2010 (except 2000 and 2002 when fieldwork was much reduced), the study area was thoroughly searched for active nest sites. Active sites were identified early in the breeding season (early May) and revisited to ring chicks at 12 (± 2 days) days posthatch, that is, 6–10 days before fledging. First brood RS, defined as the number of first brood chicks ringed (RS_FL_), was therefore measured at every nest site monitored during the long-term study. Active nest sites that failed (i.e., where eggs were laid but no chicks survived to ringing) were assigned RS_FL_ = 0. Nest sites in which no eggs were laid in a particular year were excluded from the dataset. Some nest sites were reused across multiple years while others were used only once (median: 3 years, interquartile range: 1–6 years).

In 6 years (1985, 1996, 2007, 2008, 2009, and 2010), all nest sites were monitored throughout the spring and early summer to document the occurrence and success of second broods. All nest sites were visited to ring second brood chicks at 12 (± 2 days) days posthatch, as for first broods. Each nest site's seasonal RS (RS_TOT_) was calculated as the total number of chicks ringed across the whole season. RS_TOT_ was therefore measured for individual nest sites rather than individual adult starlings.

### Spatial clusters

As in many natural populations there are no obviously distinct subpopulations within Fair Isle, starling nest sites are distributed unevenly with no clear demarcation of habitat patches or other ecological or environmental variables evident to human observers that could potentially influence RS. Moreover, nest sites do not fall into clear discrete areas; some sites are isolated, others are tightly bunched or linearly distributed (Fig. 2). However, despite this lack of distinct subdivision, the population is not panmictic. Natal dispersal and foraging movements are highly spatially restricted. Specifically, median natal dispersal distance was only 771 m across 88 individuals that were color-ringed as chicks that subsequently recruited, and field observations of color-ringed individuals show that breeding season foraging movements are highly restricted. We therefore hypothesized that RS would also vary on a small-scale spatial scale within the population.

In the absence of clear a priori ecological or environmental divisions, we used hierarchical cluster analysis (HCA, Appendix S1) to define discrete spatial groupings (hereafter “clusters”) of nest sites at multiple spatial scales without imposing arbitrary a priori divisions. The HCA used nest site locations, and hence the population's physical structure, to define spatial clusters based on linear distances among sites. Each individual nest site initially forms a unique “cluster”. These clusters are then fused hierarchically based on a distance algorithm until one cluster containing every site remains (Appendix S1). The “scalar distance” measures the distinctness and stability of resulting clusters (Appendix S1). Clustering by linear distance is likely to be biologically meaningful in the context of starling RS, because starlings are nonterritorial breeders and forage in loose flocks with proximate conspecifics, breeders from adjacent nest sites are likely to experience similar environmental conditions (see Discussion).

Nest site locations were recorded using handheld GPS, or noted on a detailed map (Fig. 2). Sites whose locations were less precisely recorded were not included in the HCA but were included in analysis of RS when their approximate location fell clearly within a defined cluster.

Across 286 nest sites with precisely known locations, the median separation was 1594 m (range 1–4912 m). The HCA identified four sets of clusters that remained stable over the largest scalar distances, containing seven, four, three, and two clusters, respectively, and thereby defined a hierarchy of four spatial scales based on the system's physical structure (Fig. 2, Appendix S1). These four sets of clusters were more stable than equivalent clusters defined when the HCA was run again on randomized nest site locations (Appendix S1). Even smaller scales were not considered because clusters became relatively unstable, and the number of observations of RS within each cluster would inevitably have been too small for meaningful analysis of spatial variation (Appendix S1).

The population's physical structure did not change markedly over the long-term study. Active nest sites were present in every spatial cluster in almost every year at all four spatial scales. The only exception was one cluster at the seven-cluster scale, which only contained four sites; these were inactive in four nonconsecutive and widely spaced years (1980, 1993, 2001, and 2010).

### First brood reproductive success: long-term data

The long-term data describing first brood RS (RS_FL_) covered 29 years (1980 to 2010, excluding 2000 and 2002). To test whether RS_FL_ varied at small spatial scales within Fair Isle and whether the pattern of spatial variation was consistent across years (thereby answering questions 1 and 2), we used generalized linear models (GLMs) with Poisson error structures and log link functions to test for main effects of cluster and year on RS_FL_, and for cluster by year interactions. We fitted these models at each of the four spatial scales defined by HCA to test whether the spatial scale considered affected the estimated magnitude of spatial or spatio-temporal variation (question 4). We fitted three spatial models at all four spatial scales: a model with spatial cluster as a categorical fixed effect; an additive model with spatial cluster plus year (also modeled as a categorical fixed effect); and a full model with a year by spatial cluster interaction. This interaction term enabled us to test whether the pattern of spatial variation was stable over time. The absence of a significant interaction would indicate that patterns of spatial variation were stable, for example, if some clusters had consistently higher RS than others. Finally, we also fitted a single nonspatial model that included year only. This gave 13 models: three spatial models at each of the four spatial scales and one nonspatial model including year only (Table 1). To assess whether multiple observations of RS_FL_ from individual nest sites could be deemed independent, we fitted generalized linear mixed models with random nest site effects. The 95% prediction intervals for nest site effects all overlapped zero and estimates of fixed effects did not differ substantially from GLMs. GLMs are therefore presented for simplicity.

**Table 1:** Generalized linear models explaining variation in first brood reproductive success (RS_FL_) over 29 years, total seasonal reproductive success (RS_TOT_) over 6 years, and first brood reproductive success (RS_FS_) over the same 6 years. Variation with year and spatial cluster was modeled across four spatial scales. “+”and “*”indicate additive and interactive effects. AIC and residual degrees of freedom (df) are shown for each model. ΔAIC shows the increase in AIC relative to the model with the lowest AIC within each spatial scale (Scale ΔAIC) or across all scales (Global ΔAIC)

	RS_FL_				RS_TOT_				RS_FS_			
	AIC	Residual df	Scale ΔAIC	Global ΔAIC	AIC	Residual df	Scale ΔAIC	Global ΔAIC	AIC	Residual df	Scale ΔAIC	Global ΔAIC
Seven-cluster scale												
Cluster + Year	**7540.8**	**2014**	**0.0**	**3.8**	**1828.2**	**438**	**0.0**	**7.7**	**1652.6**	**438**	**0.0**	**4.9**
Year	7545.0	2020	4.2	8.0	1848.2	444	20.0	27.7	1653.7	444	1.1	6.0
Cluster	7630.9	2042	90.1	93.9	1862.9	443	34.7	42.4	1672.3	443	19.7	24.6
Four-cluster scale												
Cluster*Year	7642.4	1933	105.4	105.4	**1822.1**	**426**	**0.0**	**1.6**	1663.7	426	15.8	16.0
Cluster + Year	**7537.0**	**2017**	**0.0**	**0.0**	1822.2	441	0.1	1.7	**1647.9**	**441**	**0.0**	0.2
Year	7545.0	2020	8.0	8.0	1848.2	444	26.1	27.7	1653.7	444	5.8	6.0
Cluster	7626.0	2045	89.0	89	1857.9	446	35.8	37.4	1667.6	446	19.7	19.9
Three-cluster scale												
Cluster*Year	7606.9	1962	64.8	69.9	**1820.5**	**432**	**0.0**	**0.0**	1656.4	432	7.8	8.7
Cluster + Year	**7542.1**	**2018**	**0.0**	**5.1**	1830.8	442	10.3	10.3	**1648.6**	**442**	**0.0**	**0.9**
Year	7545.0	2020	2.9	8.0	1848.2	444	27.7	27.7	1653.7	444	5.1	6.0
Cluster	7631.8	2046	89.7	94.8	1864.6	447	44.1	44.1	1666.9	447	18.3	19.2
Two-cluster scale												
Cluster*Year	7565.1	1991	24.8	28.1	**1823.4**	**438**	**0.0**	**2.9**	1648.4	438	0.7	0.7
Cluster + Year	**7540.3**	**2019**	**0.0**	**3.3**	1834.5	443	11.1	14.0	**1647.7**	**443**	**0.0**	**0.0**
Year	7545.0	2020	4.7	8.0	1848.2	444	24.8	27.7	1653.7	444	6.0	6.0
Cluster	7630.3	2047	90.0	93.3	1867.2	448	43.8	46.7	1665.4	448	17.7	17.7

Bold indicates the best-supported model within each spatial scale. Shading indicates the best-supported models for RS_FL_, RS_TOT_, and RS_FS_ across all four spatial scales. The full cluster*year model was not run at the seven-cluster scale because one cluster was unoccupied in some years.

We used Akaike information criteria (AIC) to identify the best-supported model within and among spatial scales. AIC allow comparison of non-nested models such as those fitted across different spatial scales. The best-supported model was defined as that with the lowest AIC, with models separated by AIC < 2 deemed similarly well supported. This approach is more robust than identifying “minimum adequate models”, especially when multiple models may be similarly well supported (Burnham and Anderson 2002; Whittingham et al. 2006). Mean brood sizes for years and clusters were predicted by back-transforming model estimates.

### Total seasonal reproductive success

To test whether patterns of spatio-temporal variation in first brood RS (RS_FL_) are similar when season-long RS is considered (RS_TOT_, question 3), we estimated among-year and among-cluster variation in RS_TOT_ using a further set of 13 GLMs (as for RS_FL_) across data from the 6 years when RS_TOT_ was measured.

### First brood reproductive success: short-term data

An additional aim was to determine whether the pattern of spatio-temporal variation in RS_TOT_ would have been accurately described if only first brood RS had been measured. We therefore fitted the same set of 13 GLMs across first brood data from the 6 years in which RS_TOT_ was measured (hereafter “RS_FS_”). RS_FL_ and RS_FS_ both measure the number of chicks ringed per first brood but span long and short datasets, respectively. The best-supported models within and among spatial scales were identified using AIC as above.

All GLMs were validated by plotting residuals against fitted values and explanatory variables (Zuur et al. 2010). Correlograms of model residuals showed that little spatial autocorrelation remained after modeling spatial cluster (Appendix S2). Randomization of nest sites across clusters was used to verify that any spatial variation observed at smaller spatial scales did not simply reflect increased sampling variance (Appendix S3). Statistical analysis, cluster analysis, and correlograms were produced using R packages stats and cluster and spatial, respectively (Venables and Ripley 2002; Maechler et al. 2011; R Development Core Team 2011).

### Frequency of second broods

The low frequency of second broods (see Results) meant that variation in second brood RS could not be analyzed in the same way as RS_FL_, RS_TOT_, and RS_FS_. The proportion of active first brood nest sites within each cluster that contained a successful second brood (pRS_2_) was therefore calculated to quantify the contribution of second broods to RS_TOT_ across each spatial scale.

## Results

### Spatial clusters

The mean (±1 SD) distances between nest sites within clusters were 258 ± 153 m, 423 ± 51 m, 661 ± 216 m, and 816 ± 426 m at the seven-, four-, three-, and two-cluster scales, respectively. All clusters were therefore small relative to a starling's potential mobility. Median and mean numbers of active nest sites per cluster per year are shown in Appendix S4.

### First brood reproductive success: long-term data

**Figure 3:**
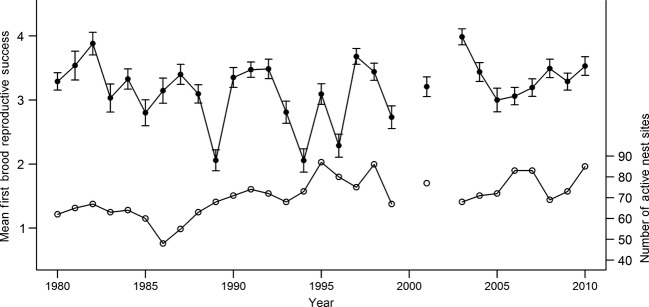
Predicted mean first brood reproductive success (RS_FL_ left axis, filled circles) ±1 SE of European starlings on Fair Isle. The number of active nest sites on the island is also shown (right axis, open circles) in each year (excluding 2000 and 2002).

There were 2049 observations of RS_FL_ over 29 years (mean 71 per year, range 48–87). Mean RS_FL_ across all years and nest sites was 3.2 and ranged from 2.1 in 1994 to 4.0 in 2003 (Fig. 3).

Models that included year effects were strongly supported (ΔAIC > 80, Table 1), showing that mean RS_FL_ varied among years. There was also strong support for models that included cluster, showing that mean RS_FL_ varied among spatial clusters (Table 1, Fig. 2). Within each of the four spatial scales, the best-supported model contained additive effects of cluster as well as year (ΔAIC > 2, Table 1). The maximum difference in predicted mean RS_FL_ between clusters was greater at the smaller seven- and four-cluster scales (0.5 and 0.4 chicks per nest site, respectively) than at the larger three- and two-cluster scales (0.2 chicks per site, Fig. 2). The pattern of spatial variation across Fair Isle did not show a clear trend from south to north (Fig. 2). Models that included a year by cluster interaction were not well supported at any of the four spatial scales (ΔAIC > 20, Table 1) even after excluding the cluster at the seven-cluster scale that was unoccupied in 4 years (cluster 2, ΔAIC > 170).

When all 13 models fitted across all four spatial scales were compared, the best-supported model included additive effects of year and cluster at the four-cluster scale, but no year by cluster interaction (Table 1, ΔAIC > 3). The most parsimonious model therefore included four spatial clusters and showed that RS_FL_ varied among years and among clusters. The relative lack of support for the four-cluster model that included a year by cluster interaction indicates that the pattern of among-cluster variation in RS_FL_ was stable across years.

### Total seasonal reproductive success

**Figure 4:**
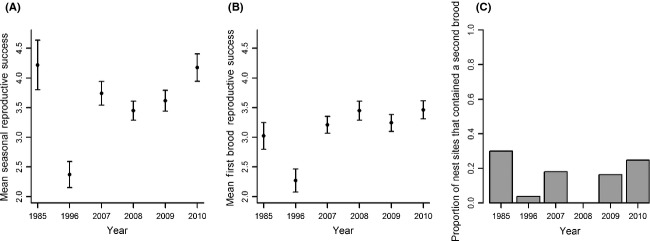
Mean predicted total seasonal reproductive success (RS_TOT_, panel A) ±1 SE of European starlings on Fair Isle which included multiple broods across the whole breeding season. First brood reproductive success (RS_FS_, panel B) ±1 SE and the proportion of first brood nest sites that contained a second brood (pRS_2_, panel C) across the 6 years in which RS_TOT_ were measured.

There were 450 observations of RS_TOT_ over 6 years (mean 75 per year, range 60–85). Mean RS_TOT_ across all years and nest sites was 3.5 and ranged from 2.4 in 1996 to 4.2 in 1985 (Fig. 4).

**Figure 5:**
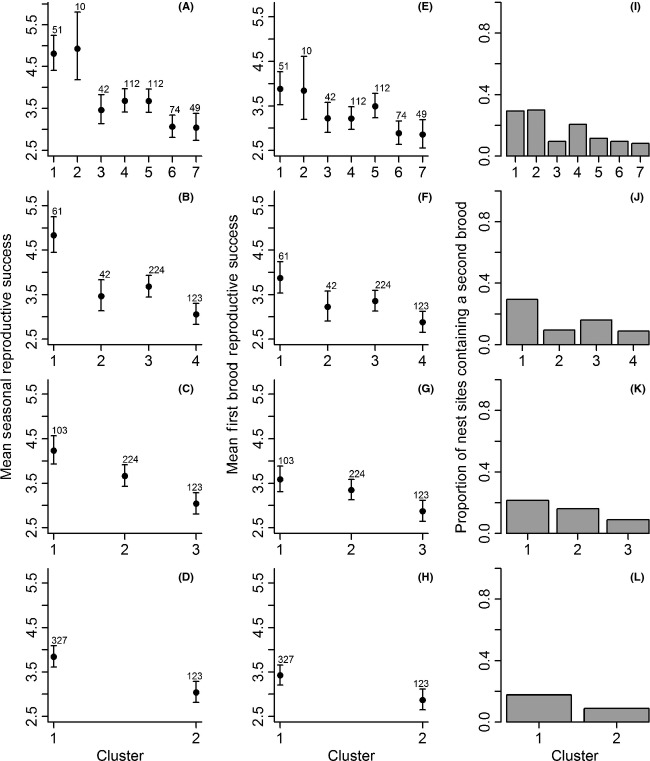
Mean predicted total seasonal reproductive success, (RS_TOT_, panels A–D) ±1 SE of European starlings on Fair Isle which included multiple broods across the whole breeding season. First brood reproductive success is also shown, (RS_FS_, panels E–H) ±1 SE as well as the proportions of first brood nest sites that contained a second brood (pRS_2_, panels I–L) for clusters defined at four spatial scales. Clusters are numbered north to south (see Fig. 2). Predictions are shown for an average year. Sample size (i.e., total number of observations of RS per cluster across years) is shown above predicted means.

Models that included year effects were strongly supported (ΔAIC > 9, Table 1) showing that mean RS_TOT_ varied among years. There was also strong support for models that included cluster and showing that mean RS_TOT_ varied among spatial clusters (Fig. 5, Table 1). Within each of the four spatial scales, the best-supported model contained additive effects of cluster as well as year (ΔAIC > 13, Table 1). The maximum difference in predicted mean RS_TOT_ between clusters was greater at the smaller seven- and four-cluster scales (1.9 and 1.8 chicks per nest site, respectively) than at the larger three- and two-cluster scales (1.2 and 0.8 chicks per site, respectively, Fig. 5). Predicted RS_TOT_ broadly decreased from north to south; northernmost clusters had substantially greater RS_TOT_ than southernmost clusters, particularly at the seven- and four-cluster scales (Fig. 5).

Models that included the year by cluster interaction were strongly supported at the three- and two-cluster scales (ΔAIC > 10), marginally supported at the four-cluster scale (ΔAIC = 0.1) but not supported at the seven-cluster scale, even after excluding the cluster that was unoccupied in 2010 (cluster 2, ΔAIC = 4.8).

When all 13 models fitted across all four spatial scales were compared, the best-supported model included additive effects of year and cluster and their interaction at the three-cluster scale (Table 1). This model was marginally better supported than the analogous model at the four-cluster scale (ΔAIC < 2) and substantially better supported than the best models at the seven- and two-cluster scales (all ΔAIC > 2, Table 1). The most parsimonious model therefore included three spatial clusters and showed that RS_TOT_ varied among cluster–years rather than solely among years and clusters independently, indicating that the pattern of spatial variation was not stable across years.

### First brood reproductive success: short-term data

Across the 450 nest-site-years included in the analysis of RS_TOT_, RS_FS_ varied substantially among years, although over a smaller range than RS_TOT_ (Fig. 4, Table 1). Mean RS_FS_ across all years and nest sites was 3.1 and ranged from 2.3 in 1996 to 3.5 in 2010 (Fig. 4).

Models that included year effects were strongly supported (ΔAIC > 10, Table 1) showing that mean RS_FS_ varied among the 6 years in which RS_TOT_ was recorded. Models that included cluster were also strongly supported, showing that RS_FS_ varied among spatial clusters. The best-supported model within each of the four spatial scales included additive effects of cluster and year, although this model was only marginally better supported than the year-only model at the seven-cluster scale (ΔAIC = 1.1, Table 1). RS_FS_ showed a qualitatively similar pattern of spatial variation to RS_TOT_ although the magnitude of variation was smaller (Fig. 5, panels A–D and E–H).

When all 13 models fitted across all four spatial scales were compared, the best-supported model for RS_FS_ included additive effects of year and cluster at the two-cluster scale, although this model was only marginally better supported than the analogous models at the three- and four-cluster scales (ΔAIC = 0.9 and 0.2, respectively, Table 1). Models that included a year by spatial cluster interaction were not well supported (ΔAIC > 6, Table 1), even after excluding the cluster at the seven-cluster scale that was unoccupied in 2010 (cluster 2, ΔAIC > 26).

### Frequency of second broods

Successful second broods were uncommon: The proportions of active first brood nest sites that contained a successful second brood (pRS_2_) varied across the 6 years when RS_TOT_ was recorded (range 0–0.30, mean 0.15, Fig. 4). The pattern of spatial variation in pRS_2_ was qualitatively similar to that in RS_TOT_ and RS_FS_; pRS_2_ was high in northern clusters and much lower in southern clusters (Fig. 5, panels I–L, for example, 0.30 in the north compared to 0.09 in the south at the four-cluster scale). Quantitatively, second broods substantially increased RS_TOT_ in the northern clusters, especially for clusters defined at small spatial scales. At the four-cluster scale, the difference in RS_FS_ between the northern and southernmost clusters was 1.0 chick per nest site, but the difference in RS_TOT_ was 1.8 chicks per nest site (Fig. 5, panels A and E).

## Discussion

Empiricists and theoreticians often assume that demography is spatially homogeneous within populations that are not clearly subdivided into discrete patches or obviously distinct habitat types (Coulson et al. 1999). Contrary to this assumption, we detected considerable spatial variation in first brood (RS_FL_) and total seasonal (RS_TOT_) reproductive success among small spatial clusters defined by the physical locations of nest sites within a single starling population. RS_FL_ varied substantially among years and spatial clusters, and the pattern of spatial variation was consistent across years. The magnitudes of spatial and temporal variation in RS_TOT_ were even greater than in RS_FL_, and moreover, the pattern of spatial variation was no longer consistent across years. Furthermore, the estimated magnitudes of spatial variation in RS_FL_ and RS_TOT_ depended on the spatial scale at which clusters were defined, being greater at smaller scales. We therefore demonstrate substantial spatio-temporal variation in a major demographic rate across very small spatial scales within an apparently contiguous starling population and show that the estimated magnitude of such variation depends on the measure of reproductive success and the choice of spatial scale.

### Spatial clusters

Identifying appropriate spatial scale(s) over which to measure demographic variation is difficult. In the absence of obvious spatial subdivisions, such as discrete subpopulations or habitat patches, analysts sometimes impose arbitrary divisions that are unrelated to the specific spatial nature of the system and may consequently draw spurious conclusions (Wheatley and Johnson 2009). For example, Steen and Haydon (2000) showed that if census scales were smaller than twice natal dispersal distance, *λ* for snowshoe hares (*Lepus americanus*) was underestimated, particularly at low densities. However, spatial variation in all ecological and environmental variables that could influence demography (potentially including resources, microclimate, topography, predators, parasites, and pollutants) will rarely be measured, meaning that corresponding spatial divisions cannot be defined. We used HCA to define objective spatial clusters at multiple spatial scales based on the physical locations of nest sites and hence the physical structure of the study population. The HCA identified stable clusters at four spatial scales. Similarly, stable clusters were not evident when nest site locations were randomized (Appendix S1). Clustering by nest site location is biologically relevant for starlings because they are semi-colonial and nonterritorial; they defend only the immediate nest site (Feare 1984) and preferentially forage with conspecifics from adjacent nest sites (Vásquez and Kacelnik 2000). This may be because individuals use the foraging success of others to assess patch quality (Templeton and Giraldeau 1996) and increase food intake at higher densities (Fernandez-Juricic 2004). Such spatial foraging associations could potentially translate into spatial variation in reproductive success. Furthermore, as starlings occupy fixed cavity nest sites, the population's physical structure remained broadly similar across years. Such ecological knowledge is essential when defining appropriate spatial groupings and scales for any focal species. For instance, grouping by nest site location may be inappropriate for territorial species where individual foraging ranges do not overlap and environment varies among adjacent territories.

### Spatio-temporal variation in first brood reproductive success

RS_FL_ varied substantially among the 29 study years, from 2.1 to 4.0 ringed chicks per attempt. This range of 1.9 chicks per nest site equates to 61% of the grand mean RS_FL_ of 3.2. This range is substantial, but not remarkable compared to that observed in other bird species. Selected studies that measured RS across ≥5 years observed ranges of 1.2 chicks per attempt in western bluebirds (5 years, *Sialia mexicana,* Keyser et al. 2004), 1.2 in choughs (20 years, Reid et al. 2004); 1.2 in black-legged kittiwakes (15 years, *Rissa tridactyla*, Murphy et al. 1991); and 1.7 in goshawks (10 years, *Accipiter gentilis*, Mcclaren et al. 2002).

RS_FL_ also varied substantially among spatial clusters at all four defined spatial scales. For example, at the best-supported four-cluster scale, mean predicted brood sizes ranged from 2.9 to 3.4 chicks per attempt, equating to ca. 16% of the grand mean RS_FL_ of 3.2. This magnitude of spatial variation in RS is comparable to that observed across much larger spatial scales in other species. In European blackbirds (*Turdus merula*) and song thrushes (*Turdus philomelos*), for example, the largest differences in RS between 10 km^2^ plots across the UK were 0.6 and 0.8 chicks, respectively (Paradis et al. 2000). Arlt et al. (2008) and Blondel (2007) observed differences of 1.1 and 3.6 chicks per brood between entirely different habitats in Wheatears (*Oenanthe oenanthe*) and Blue Tits (*Cyanistes caeruleus*), respectively. The magnitude of spatial variation in RS_FL_ observed within Fair Isle's starling population is notable given the very small overall size of the study area (ca.5x2.5 km) and broadly similar grassland habitat. Very small-scale spatial variation in demography cannot, therefore, necessarily be assumed to be negligible within apparently continuous landscapes.

Furthermore, the magnitude of among-cluster variation in RS_FL_ was greater when measured across smaller spatial clusters. The largest predicted difference among clusters at the seven-cluster scale was more than twice that at the two-cluster scale (0.5 vs. 0.2 chicks per nest site). Even the largest scale that we considered (two clusters) might typically be interpreted as “small-scale”. The mean distance between nest sites within clusters was only 816 m (±426 SD), orders of magnitude shorter than a starling's dispersal capability (Feare 1984). However, even this scale was large enough to mask the spatial variation in RS_FL_ that was evident at the smaller three-, four-, and seven-cluster scales (Fig. 2). The choice of spatial scale therefore substantially affected the estimated magnitude of spatial variation in a key demographic rate. Simulations confirmed that these patterns and conclusions do not simply reflect increased stochastic variance in smaller spatial scales, for example, because smaller clusters contain fewer nest sites and hence fewer observations of RS_FL_ (Appendix S3). Indeed, because our analyses used multiple years of RS data, sample sizes were large even when clusters contained relatively few nest sites.

There was no evidence of spatio-temporal variation in RS_FL_, defined as a year by cluster interaction, even at the largest two-cluster spatial scale where statistical power was substantial. Spatial variation in RS_FL_ was therefore relatively consistent over the 29-year study (Appendix S4). This stability implies that even relatively small differences in mean RS_FL_ between clusters could ultimately affect population dynamics, especially if there was correlated variation in other demographic rates (Saether and Bakke 2000). All else being equal, the more productive (northern) clusters might then act as sources, exporting recruits to less productive (southern) clusters. Such consistent spatial variation may also help to regulate population size, if individuals move into areas with higher productivity at smaller population sizes (Rodenhouse et al. 1997).

### Spatio-temporal variation in total seasonal reproductive success

Many studies rely on monitoring a single breeding attempt to measure RS because following multiple attempts can be difficult and time-consuming (Siriwardena et al. 2000; Thompson et al. 2001; Cornulier et al. 2009; Sim et al. 2011). However, variation in first brood RS may not accurately predict variation in total seasonal RS because individuals may trade-off producing large first broods against producing subsequent broods, or environmental variation may mean that some individuals can produce multiple successful broods. We therefore quantified spatio-temporal variation in RS_TOT_ over 6 years and assessed whether observed variation would have been correctly estimated had only first brood data (RS_FS_) been collected.

RS_TOT_ varied substantially among the 6 years in which all broods were monitored, from 2.4 to 4.2 chicks per nest site. The magnitude of among-year variation in RS_TOT_ was greater than that in first brood RS (RS_FS_) measured over the same 6 years (range of 2.3–3.5 chicks). This was primarily because the probability of double brooding (pRS_2_) varied from 0 to 0.30. Furthermore, including data from multiple breeding attempts increased the estimated magnitude of spatial variation in RS. RS_TOT_ varied from 3.0 to 4.9 chicks per nest site at the seven-cluster scale, while RS_FS_ varied from 2.9 to 3.9 chicks per site at this same scale. This occurred because RS_FS_ and pRS_2_ showed qualitatively similar patterns of spatial variation (Fig. 5). Specifically, nest sites in the northernmost cluster at the seven-cluster scale gained 0.9 chicks per year because 30% of sites contained second broods on average. In contrast, nest sites in the southernmost cluster gained only 0.2 chicks per year on average because only 9% of sites contained second broods. These patterns do not match expectation assuming a trade-off between first brood and second brood RS. They may instead reflect small-scale spatial variation in resources or other determinants of RS.

Quantifying RS_TOT_ not only altered the estimated magnitudes of temporal and spatial variation in RS, but also altered estimates of spatio-temporal variation. There was no evidence of spatio-temporal variation, manifested as a significant year by cluster interaction, in either RS_FS_ or RS_FL_. In contrast, analysis of RS_TOT_ showed significant interactions at the three- and two-cluster scales. These interactions stem from the spatio-temporal variation in pRS_2_ (Fig. 4); high pRS_2_ in some clusters in some years increased spatio-temporal variation in RS_TOT_ compared to RS_FS_ and RS_FL_. Previous studies on passerines have also observed among-year variation in the frequency of second broods; the percentages of black throated blue warblers (*Dendroica caerulescens*) that were double-brooded varied from 0 to 87% over 7 years (Nagy and Holmes 2005). Such among-year variation means that season-long data need to be collected over multiple years in order to infer the consequences of multiple brooding for spatio-temporal variation in *λ*.

### Population consequences

Very small-scale spatial variation in RS, such as we observed in Fair Isle's starlings, could potentially occur in other mainland and island populations and influence population dynamics in multiple ways. However, these consequences depend on the causal mechanisms, the degree to which variation in other demographic rates is spatially correlated, and the degree to which dynamics are intrinsic to any focal population or driven by larger scale immigration or emigration. Internal population regulation is predicted if individuals preemptively settle in areas of intrinsically high productivity (Rodenhouse et al. 1997; Sergio and Newton 2003). Such intrinsic variation in RS could be caused by small-scale spatial variation in foraging conditions or nest site microclimate (as previously observed in starlings, Reid et al. 2000; Smith and Bruun 2002), or other environmental factors. However, RS could be high in certain areas because of low breeding density (for example, due to nest site limitation) rather than any particular environmental qualities. While the influence of habitat on RS was greater than that of density in eight populations of territorial raptors (Kruger, Chakarov, and Nielsen 2012), density might have greater effects in semi-colonial species such as the starling. Predicting the population consequences of small-scale spatial variation in RS such as we observed in Fair Isle's starlings therefore requires further consideration of both the underlying ecological mechanisms and other demographic rates, including immigration and emigration. Nevertheless, in general, our data suggest that empiricists and theoreticians should not necessarily assume that key demographic rates are spatially homogeneous at small spatial scales.

## Supporting Information

**Appendix S1.** Defining spatial clusters using hierarchical cluster analysis. **Appendix S2.** Residual spatial autocorrelation. **Appendix S3.** Spatial variation in reproductive success and sampling variance. **Appendix S4.** Spatio-temporal interactions in reproductive success.

## References

[b1] Aparicio JP, Solari HG, Bonino N (2004). Competition and coexistence in host-parasite systems: the myxomatosis case. Popul. Ecol.

[b2] Arlt D, Forslund P, Jeppsson T, Part T (2008). Habitat-specific population growth of a Farmland Bird. PLoS One.

[b3] Banda E, Blanco G (2009). Implications of nest-site limitation on density-dependent nest predation at variable spatial scales in a cavity-nesting bird. Oikos.

[b4] Blondel J (2007). Coping with habitat heterogeneity: the story of Mediterranean blue tits. J. Ornithol.

[b5] Burnham K, Anderson DR (2002). Model selection and multimodel inference: a practical information theoretic approach.

[b6] Chave J (2013). The problem of pattern and scale in ecology: what have we learned in 20 years?. Ecol. Lett.

[b7] Chivers WJ, Gladstone W, Herbert RD, Fuller MM (2014). Predator–prey systems depend on a prey refuge. J. Theor. Biol.

[b700] Cramp S, Perrins CM, Brooks DJ, Dunn E, Gillmor R, Hall-Craggs J, Hillcoat B, Hollom PAD, Nicholson EM, Roselaar EM, Seale WTC, Sellar PJ, Simmons KEL, Snow DW, Vincent D, Wallace DIM, Wilson MG (1993). Handbook of the birds of Europe, the Middle East and North Africa: the birds of the Western Palearctic.

[b8] Cornell KL, Donovan TM (2010). Effects of spatial habitat heterogeneity on habitat selection and annual fecundity for a migratory forest songbird. Landscape Ecol.

[b9] Cornulier T, Elston DA, Arcese P, Benton TG, Douglas DJT, Lambin X (2009). Estimating the annual number of breeding attempts from breeding dates using mixture models. Ecol. Lett.

[b10] Coulson T, Albon S, Guinness F, Pemberton J, Clutton-Brock T (1997). Population substructure, local density, and calf winter survival in Red Deer (*Cervus Elaphus*. Ecology.

[b11] Coulson T, Albon S, Pilkington J, Clutton-Brock T (1999). Small-scale spatial dynamics in a fluctuating ungulate population. J. Anim. Ecol.

[b12] Coulson T, Catchpole EA, Albon SD, Morgan BJ, Pemberton JM, Clutton-Brock TH (2001). Age, sex, density, winter weather, and population crashes in Soay sheep. Science.

[b13] Cowen RK, Paris CB, Srinivasan A (2006). Scaling of connectivity in marine populations. Science.

[b14] De Knegt HJ, van Langevelde F, Skidmore AK, Delsink A, Slotow R, Henley S (2011). The spatial scaling of habitat selection by African elephants. J. Anim. Ecol.

[b15] De Roos AM, Mccauley E, Wilson WG (1991). Mobility versus density-limited predator-prey dynamics on different spatial scales. Proceed. Royal Soc. London B.

[b16] Donovan TM, Thompson FR, Faaborg J, Probst JR (1995). Reproductive success of migratory birds in habitat sources and sinks. Conserv. Biol.

[b17] Evans PGH (1980).

[b19] Feare C (1984). The starling.

[b20] Fernandez-Juricic E (2004). Flock density, social foraging, and scanning: an experiment with starlings. Behav. Ecol.

[b21] Fortescue M (1999). Temporal and spatial variation in breeding success of the little penguin *Eudyptula minor* on the east coast of Australia. Marine Ornithol.

[b22] Gaillard J, Festa-Bianchet M, Yoccoz NG, Loison A, Toigo C (2000). Temporal variation in fitness components and population dynamics of large herbivores. Annu. Rev. Ecol. Syst.

[b23] Hanski I (1998). Metapopulation dynamics. Nature.

[b25] Husby A, Kruuk LEB, Visser ME (2009). Decline in the frequency and benefits of multiple brooding in great tits as a consequence of a changing environment. Proceed. Royal Soc. London B.

[b26] Johnson DM (2004). Source-sink dynamics in a temporally heterogeneous environment. Ecology.

[b27] Keyser AJ, Keyser MT, Promislow DEL (2004). Life-history variation and demography in western bluebirds (*Sialia mexicana*) in Oregon. Auk.

[b28] Krüger O, Chakarov N, Nielsen JT, Looft V, Grünkorn T, Struwe-Juhl B (2012). Population regulation by habitat heterogeneity or individual adjustment?. J. Anim. Ecol.

[b30] Levin SA (1992). The problem of pattern and scale in ecology: The Robert H. MacArthur Award Lecture. Ecology.

[b31] Maechler M, Rousseeuw P, Struyf A, Hubert M, Hornik K (2011). cluster: Cluster analysis basics and extensions.

[b32] Mcclaren EL, Kennedy PL, Dewey SR (2002). Do some Northern Goshawk nest areas consistently fledge more young than others?. Condor.

[b33] McPeek MA, Rodenhouse NL, Holmes RT, Sherry TW (2001). A general model of site-dependent population regulation: population-level regulation without individual-level interactions. Oikos.

[b34] Murphy EC, Springer AM, Roseneau DG (1991). High annual variability in reproductive success of Kittiwakes (Rissa tridactyla L.) at a Colony in Western Alaska. J. Anim. Ecol.

[b35] Nagy LR, Holmes RT (2005). To double-brood or not? Individual variation in the reproductive effort in black-throated blue warblers (Dendroica caerulescens). Auk.

[b36] Nystrand M, Griesser M, Eggers S, Ekman J (2010). Habitat-specific demography and source-sink dynamics in a population of Siberian jays. J. Anim. Ecol.

[b37] Orians GH, Wittenberger JF (1991). Spatial and temporal scales in habitat selection. Am. Nat.

[b38] Ozgul A, Armitage KB, Blumstein DT, Oli MK (2006). Spatiotemporal variation in survival rates: implications for population dynamics of yellow-bellied marmots. Ecology.

[b39] Paradis E, Baillie SR, Sutherland WJ, Dudley C, Crick HQP, Gregory RD (2000). Large-scale spatial variation in the breeding performance of song thrushes *Turdus philomelos* and blackbirds *T. merula* in Britain. J. Appl. Ecol.

[b40] Pulliam HR (1988). Sources, sinks, and population regulation. Am. Nat.

[b41] Pulliam HR, Danielson BJ (1991). Sources, sinks, and habitat selection: a landscape perspective on population dynamics. Am. Nat.

[b42] R Development Core Team (2011). R: A language and environment for statistical computing.

[b43] Reid JM, Monaghan P, Ruxton GD (2000). Resource allocation between reproductive phases: the importance of thermal conditions in determining the cost of incubation. Proceed. Royal Soc. London B.

[b44] Reid JM, Bignal EM, Bignal S, McCracken DI, Monaghan P (2004). Identifying the demographic determinants of population growth rate: a case study of red-billed choughs *Pyrrhocorax pyrrhocorax*. J. Anim. Ecol.

[b45] Reid JM, Bignal EM, Bignal S, McCracken DI, Monaghan P (2006). Spatial variation in demography and population growth rate: the importance of natal location. J. Anim. Ecol.

[b46] Rodenhouse NL, Sherry TW, Holmes RT (1997). Site-dependent regulation of population size: a new synthesis. Ecology.

[b47] Russell JC, Ruffino L (2011). The influence of spatio-temporal resource fluctuations on insular rat population dynamics. Proceed. Royal Soc. London B.

[b48] Saether B-E, Bakke O (2000). Avian life history variation and contribution of demographic traits to the population growth rate. Ecology.

[b49] Saether B-E, Ringsby TH, Bakke O, Solberg EJ (1999). Spatial and temporal variation in demography of a house sparrow metapopulation. J. Anim. Ecol.

[b50] Sandel B (2014). Towards a taxonomy of spatial scale-dependence. Ecography.

[b51] Sandel B, Smith AB (2009). Scale as a lurking factor: incorporating scale-dependence in experimental ecology. Oikos.

[b52] Saracco JF, Royle JA, DeSante DF, Gardner B (2010). Modeling spatial variation in avian survival and residency probabilities. Ecology.

[b53] Sergio F, Newton IAN (2003). Occupancy as a measure of territory quality. J. Anim. Ecol.

[b54] Sim IMW, Rebecca GW, Ludwig SC, Grant MC, Reid JM (2011). Characterizing demographic variation and contributions to population growth rate in a declining population. J. Anim. Ecol.

[b55] Siriwardena GM, Baillie SR, Crick HQP, Wilson JD (2000). The importance in the breeding performance of seed-eating birds in determining their population trends on farmland. J. Appl. Ecol.

[b56] Smith HG, Bruun M (2002). The effect of pasture on starling (*Sturnus vulgaris*) breeding success and population density in a heterogeneous agricultural landscape in southern Sweden. Agric. Ecosyst. Environ.

[b57] Sonsthagen SA, McClaren EL, Doyle FI, Titus K, Sage GK, Wilson RE (2012). Identification of metapopulation dynamics among Northern Goshawks of the Alexander Archipelago, Alaska, and Coastal British Columbia. Conserv. Genet.

[b58] Steen H, Haydon D (2000). Can population growth rates vary with the spatial scale at which they are measured?. J. Anim. Ecol.

[b59] Sutherland WJ, Freckleton RP, Godfray HCJ, Beissinger SR, Benton T, Cameron DD (2013). Identification of 100 fundamental ecological questions. J. Ecol.

[b60] Talley TS (2007). Which spatial heterogeneity framework? Consequences for conclusions about patchy population. Ecology.

[b61] Templeton JJ, Giraldeau L-A (1996). Vicarious sampling: the use of personal and public information by starlings foraging in a simple patchy environment. Behav. Ecol. Sociobiol.

[b62] Thomas CD, Kunin WE (1999). The spatial structure of populations. J. Anim. Ecol.

[b63] Thompson BC, Knadle GE, Brubaker DL, Brubaker KS (2001). Nest success is not an adequate comparative estimate of avian reproduction. J. Field Ornithol.

[b64] Tschumi M, Schaub M, Arlettaz R (2014). Territory occupancy and parental quality as proxies for spatial prioritization of conservation areas. PLoS One.

[b66] Vásquez RA, Kacelnik A (2000). Foraging rate versus sociality in the starling *Sturnus vulgaris*. Proceed. Royal Soc. London B.

[b67] Venables WN, Ripley BD (2002). Modern applied statistics with S.

[b68] Wheatley M, Johnson C (2009). Factors limiting our understanding of ecological scale. Ecol. Complex.

[b69] Whittingham MJ, Stephens PA, Bradbury RB, Freckleton RP (2006). Why do we still use stepwise modelling in ecology and behaviour?. J. Anim. Ecol.

[b70] Wiens JA (1989). Spatial scaling in ecology. Funct. Ecol.

[b71] Wilson S, Arcese P (2003). El Niño drives timing of breeding but not population growth in the song sparrow (*Melospiza melodia*. Proc. Natl Acad. Sci. USA.

[b72] Yeager LA, Layman CA, Allgeier JE (2011). Effects of habitat heterogeneity at multiple spatial scales on fish community assembly. Oecologia.

[b73] Zuur AF, Ieno EN, Elphick CS (2010). A protocol for data exploration to avoid common statistical problems. Methods Ecol. Evol.

