# Supplementary material for: Spatio-temporal variation in European starling reproductive success at multiple small spatial scales
Source: Ecol Evol. 2015 Jul 22;5(16):3364–77. doi: 10.1002/ece3.1615 (PMC4569032; doi:10.1002/ece3.1615)
Supplement: Supplementary file 1 [file ece30005-3364-sd1.doc]

**Spatio-temporal variation in European starling reproductive success at multiple small spatial scales**

Daisy Brickhill^1^*, Peter G. H. Evans^2^ and Jane M. Reid^1^

^1^ *Institute of Biological & Environmental Sciences, School of Biological Sciences,*

*Zoology Building, University of Aberdeen, Tillydrone Avenue, Aberdeen, AB24 2TZ, UK*

^2^ *School of Ocean Sciences, University of Bangor, Menai Bridge, Anglesey, Wales LL59 5AB*

**Appendix S1: Defining spatial clusters using hierarchical cluster analysis**

We used hierarchical cluster analysis (HCA) based on nest site locations to define spatial clusters of nest sites at a hierarchy of spatial scales. The resulting dendrogram illustrates the assignment of nest sites to spatial clusters (figure S1). Each nest site initially represents a cluster of one. Clusters are then fused hierarchically until one cluster containing every site remains. The algorithm used Euclidean distance calculated from z-standardised x and y coordinates. Weighted pair group average linkage rules were used because this method performs well whether clusters form clumps or long chains (both of which occur among the nest sites in our dataset, figure 1). Ward’s, single or complete linkage methods perform less well under these circumstances (Legendre & Legendre 1998). The scalar distance between linkage points (i.e. the range of ‘height’ values, figure S1) indicates the dissimilarity or distinctness of clusters (Everitt, Landau, & Leese 2001). The sets of clusters that were stable over the greatest range of ‘height’ values were identified. This criterion defined four sets of clusters for the current dataset (figure S1). Smaller clusters were not considered because the increasingly small sample sizes of nest sites within each cluster precluded rigorous estimation or comparison of reproductive success.

HCA is a strictly descriptive tool that identifies hierarchies of groupings across data points; it does not test the ‘significance’ of any level of clustering, and a hierarchy of clusters is always created so long as all data points are not identical. Since starling nest sites were patchily distributed across Fair Isle (see figure 1) there is, by definition, spatial structure in the system. However, to inspect the degree to which the form of the clusters identified by HCA differed from that which would have arisen given a less structured distribution of nest sites we reran the HCA after randomising nest site locations within Fair Isle. Specifically, each nest site was assigned x and y coordinates drawn from uniform distributions spanning the dimensions of Fair Isle. Comparison of the dendrograms stemming from the real versus randomised nest site locations clearly shows the stronger structure in the real data (figure S1, which shows one realisation of the randomisation). The HCA of the randomised dataset fuses into one cluster at smaller height values than the HCA of the observed dataset, indicating less distinct clusters. Furthermore, the height values used to define the two-, three-, four- and seven-cluster scales in the HCA of the real data do not define equally clear clusters when applied to the HCA of the randomised data (horizontal lines on figure S1). Specifically, the height at which the observed data shows seven clusters generates 20 poorly defined clusters in the randomised data (lowest horizontal line), while the four-, three- and two-cluster heights of the observed data generate eight, four and four clusters respectively in the randomised data. Further realisations of the randomisation produced similar patterns.


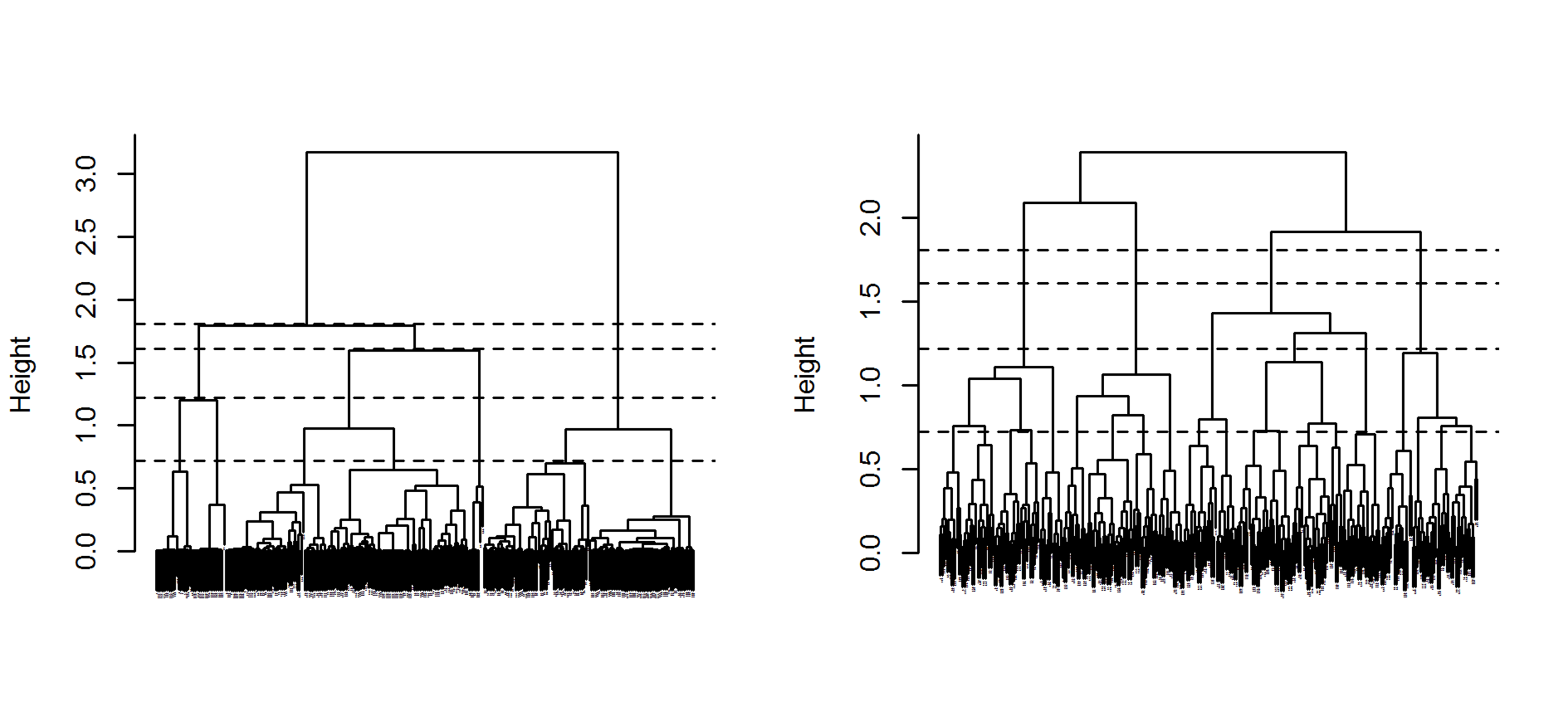


**Figure S1**. Dendrograms showing hierarchical cluster analysis (HCA) of (left) observed starling nest site locations on Fair Isle and (right) randomised nest site locations. The y-axis (height) shows the scalar distance between linkage points. The four heights (horizontal lines) that defined the most stable clusters given the real nest site locations did not define equivalent numbers of stable clusters when applied to the randomised data.

**References**

Legendre P and Legendre L (1998) *Numerical Ecology*. Elsevier B.V., Amsterdam.

Everitt BS, Landau S, Leese M (2001) *Cluster Analysis*. Arnold, London.

**Appendix S2: Residual spatial autocorrelation**

The main analyses included both year and spatial cluster as fixed effects, and in some cases year by cluster interactions. This structure should account for any temporal (among-year) auto-correlation in reproductive success. However, these analyses could potentially be biased by spatial auto-correlation in reproductive success occurring at very small spatial scales within defined spatial clusters.

To assess the degree of such spatial autocorrelation we plotted correlograms of residuals from models that included additive effects of year and cluster at each of the four spatial scales we considered. These correlograms visualise the remaining correlations among residuals from observations separated by different distances (after accounting for year and cluster). For example, positive autocorrelation between nest sites 100m apart would show as a peak at this point on the x axis. After fitting a trend surface using the residuals and spatial co-ordinates the data was divided into 300 bins, the covariance for pairs in each bin was then divided by the variance. The correlogram was fitted using the package spatial in R. As one example, figure S2 shows the correlogram for the analysis of RS_FL_ at the seven-cluster scale. No substantial residual spatial autocorrelation was evident in this analysis (figure S2), or in any other analysis.


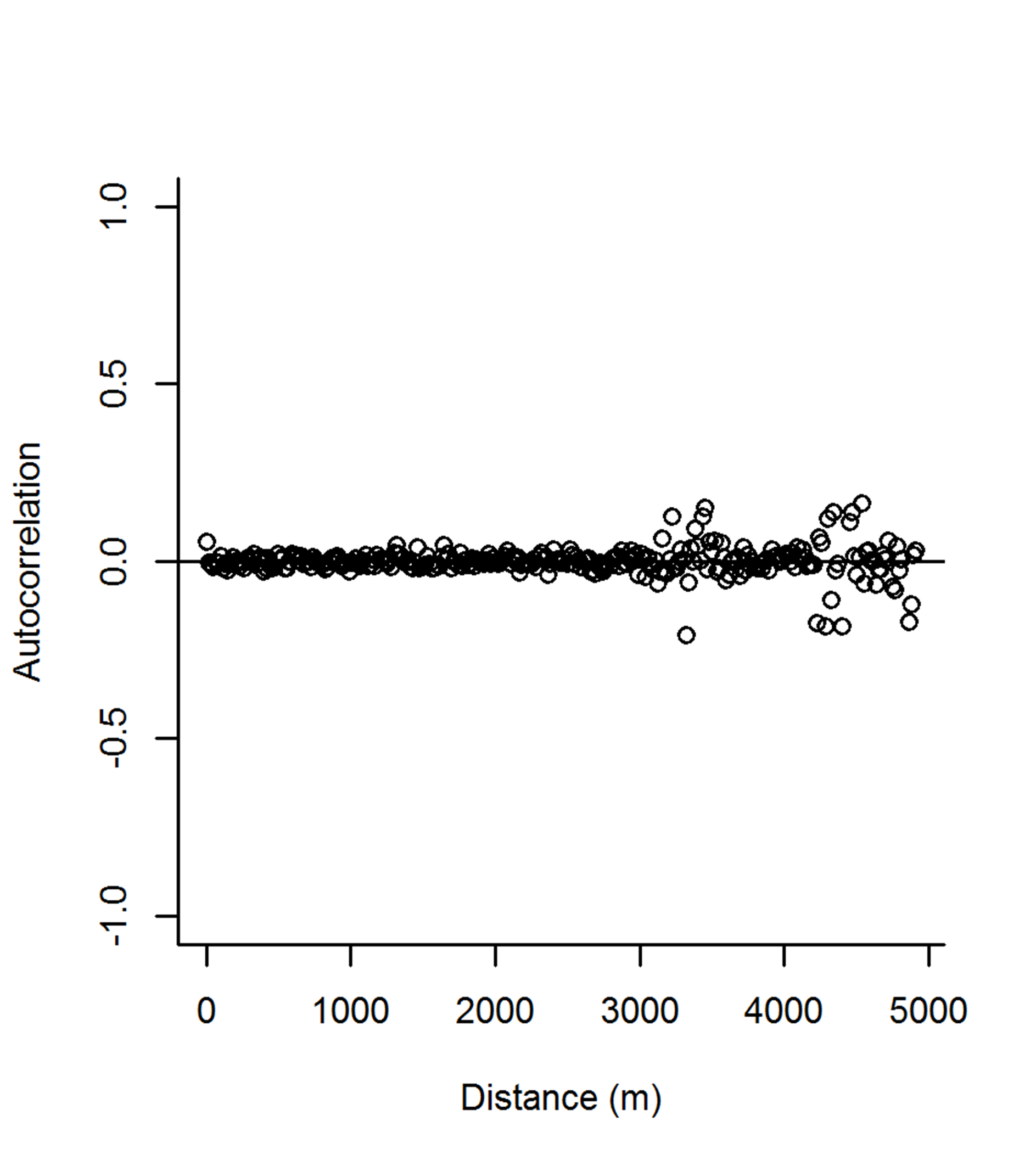


**Figure S2.** Correlogram of the autocorrelation of residuals (generated by modelling RS_FL_~cluster+year at the seven-cluster scale) as a function of distance. Points represent the spatial autocorrelation between observations of RS_FL_ at nest sites separated by varying distances after controlling for year and cluster.

**Appendix S3: Spatial variation in reproductive success and sampling variance**

Given any finite spatially explicit dataset, dividing the data into smaller spatial units will inevitably decrease the number of observations per unit and thereby potentially increase sampling variance. Statistical power to detect variation may be reduced when data are divided into smaller units.

However, our analyses used data on starling first brood reproductive success (RS_FL_) from multiple years meaning that there were numerous observations per spatial cluster, even when clusters were defined at small spatial scales (see main manuscript figures 1 & 4). However, we used simulations to further verify that the increased spatial variation in RS_FL_ observed at smaller spatial scales did not simply reflect increased sampling variance.

Each nest site was assigned to a random cluster at the seven-cluster scale without replacement (thereby retaining the original number of sites in each cluster). The models RS_FL_~cluster+year and RS_FL_~year were fitted to the randomised dataset, and the difference in AIC (ΔAIC) between the two models was recorded. This process was repeated 1000 times to generate the distribution of ΔAIC values that would arise if the difference in RS between clusters was entirely due to stochastic sampling variance. Figure S3 shows the distribution of ΔAIC values. The ΔAIC value estimated from the observed data, shown by the vertical dashed line, falls outside the randomised distribution. This simulation therefore shows that the increase in support for the model that included spatial cluster compared to the non-spatial model when both were fitted to the real data was substantially greater than expected by chance.

Since we use the AIC paradigm to compare support for multiple (non-nested) models we do not present likelihood ratio tests or associated P-values (which explicitly estimate the probability that observed variation occurred by chance given the observed sample size). However, application of these methods showed that effects of cluster on reproductive success were ‘statistically significant’ (for example, P < 0.01 for RS_FL_ at the smallest seven-cluster scale). Together, these results show that the degree of spatial variation in reproductive success that was observed at very small spatial scales is very unlikely to have arisen solely due to stochastic sampling variance.


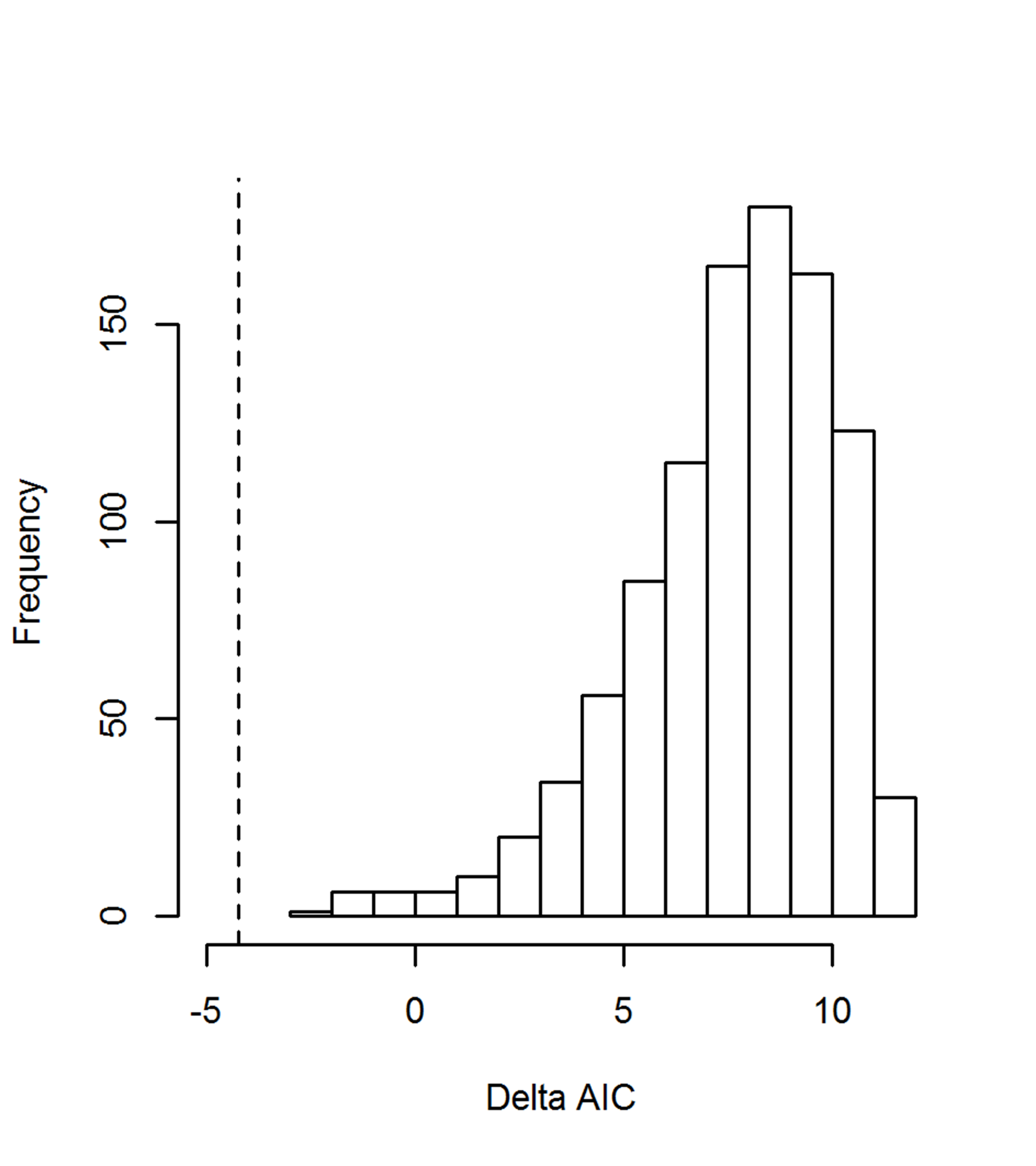


**Figure S3.** Distribution of ΔAIC values between models RS_FL_ ~cluster+year and RS_FL_ ~year fitted to randomised data at the seven-cluster scale. The dotted line shows the ΔAIC for the models fitted to the observed data.

**Appendix S4: Spatio-temporal interactions in reproductive success**

There was no support for models that included a year by spatial cluster interaction across the long-term 29 year dataset (see main paper). First-brood reproductive success (RS_FL_) varied among spatial clusters and years over the 29 year dataset (figure S4, the smallest seven-cluster scale is not visualised due to lack of clarity). These plots show that clusters typically show correlated patterns of temporal variation in RS_FL_, suggesting that the lack of an interaction does not simply reflect low statistical power. Analysis of total seasonal reproductive success (RS_TOT_) showed strong support for an interaction between year and spatial cluster at the larger three- and two-cluster scales (figure S5, panels B and C) The strong support for models that included the year by cluster interaction may be driven by 1996, which showed very different spatial patterns compared to other years (figure S5, panels A, B and C). In general, however, RS_TOT_ was higher in the northernmost clusters in most years at all three spatial scales.


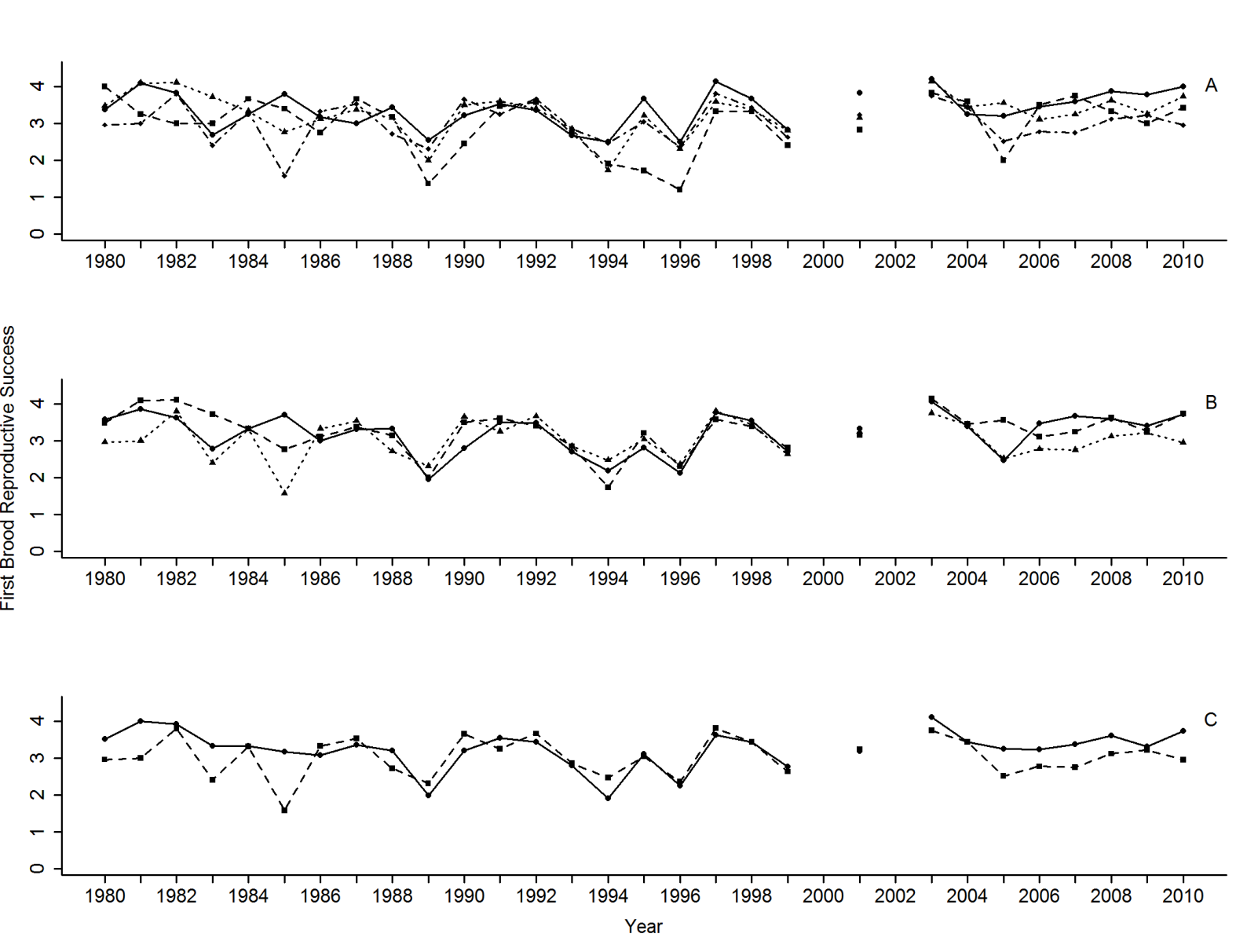


**Figure S4**. Predicted first-brood long-term reproductive success (RS_FL_) each spatial cluster at the four-, three- and two-cluster scale across 29 years (1980-2010, excepting 2000 and 2002). Across all plots cluster 1: solid line, circles, cluster 2: dashed line, squares, cluster 3: dotted line, triangles, cluster 4: dot dash line, diamonds. Clusters are numbered north to south as defined in figure 2 of the main paper.


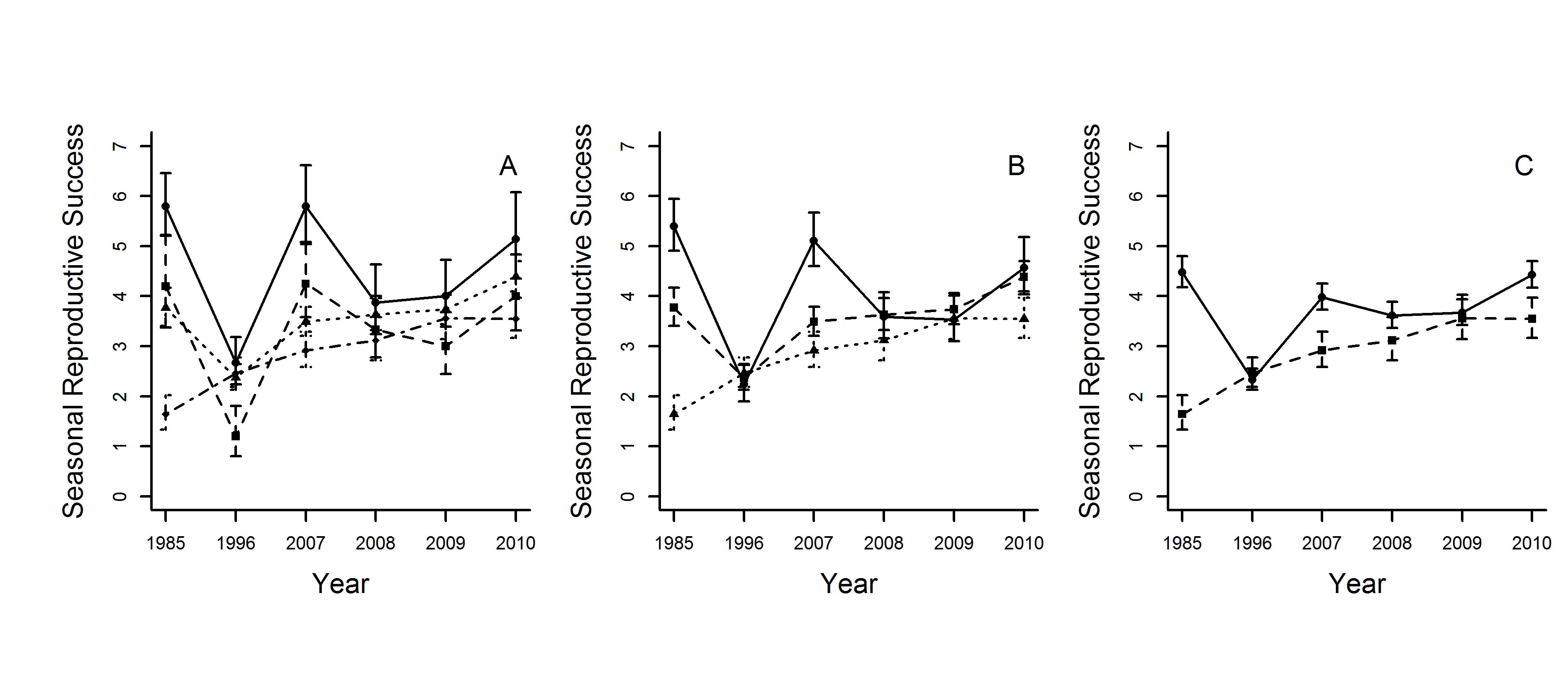


**Figure S5.** Total seasonal reproductive success (RS_TOT_) within each spatial cluster at the four- (A), three- (B) and two- (C) cluster scales across years. A: cluster 1 - solid line and circles, cluster 2 - dashed line and squares, cluster 3 - dotted line and triangles, cluster 4 - dot dash line and diamonds. B: cluster 1 - solid line and circles, cluster 2 - dashed line and squares, cluster 3 - dotted line and triangles. C: cluster 1 - solid line and circles, cluster 2 - dashed line and squares. Clusters are numbered north to south as defined in figure 1 of the main paper.

Table S1 and S2 show the median, mean and range of sample sizes used to test for the interaction between spatial cluster and year. Table S1 refers to the 29 year first-brood dataset, running from 1980 to 2010 (RS_FL_, excluding 2000 and 2002). Table S2 refers to the six year dataset (RS_TOT_ and RS_FS_, 1985, 1996, 2007, 2008, 2009 and 2010) in which both first brood and second brood reproductive success was recorded.

**Table S1.** The median, mean and range of the number of active first-brood nest sites per year in each spatial cluster at four scales over the 29 year dataset describing first-brood long-term reproductive success (RS_FL_). Clusters are numbered north to south, as defined in figure 1 of the main paper.

| **Seven-cluster scale** | **Median (mean)** | **Range** |
| --- | --- | --- |
| 1 | 8 (7.7) | 4-13 |
| 2 | 2 (2.3) | 0-4 |
| 3 | 6 (7.0) | 3-13 |
| 4 | 16 (15.1) | 7-28 |
| 5 | 18 (17.8) | 11-26 |
| 6 | 11 (10.6) | 4-20 |
| 7 | 10 (10.5) | 2-20 |
| **Four-cluster scale** |  |  |
| 1 | 10(9.7) | 5-15 |
| 2 | 6 (7.0) | 3-13 |
| 3 | 33 (32.9) | 21-49 |
| 4 | 22 (21.1) | 12-31 |
| **Three-cluster scale** |  |  |
| 1 | 17 (16.7) | 10-26 |
| 2 | 33 (32.9) | 21-49 |
| 3 | 22 (21.1) | 12-31 |
| **Two-cluster scale** |  |  |
| 1 | 52 (49.6) | 35-63 |
| 2 | 22 (21.1) | 12-31 |

**Table S2.** The median, mean and range of the number of active first-brood nest sites per year in each spatial cluster at four scales over the six year dataset describing first-brood short-term and seasonal total reproductive success (RS_FS_ and RS_TOT_). Clusters are numbered north to south, as defined in figure 1 of the main paper.

| **Seven-cluster** | **Median (mean)** | **Range** |
| --- | --- | --- |
| 1 | 8 (8.5) | 2-13 |
| 2 | 2 (2) | 0-4 |
| 3 | 7.5 (7.0) | 5-7 |
| 4 | 18.5 (18.7) | 7-28 |
| 5 | 18.5 (18.7) | 17-21 |
| 6 | 12 (12.3) | 8-10 |
| 7 | 5.5 (8.2) | 4-19 |
| **Four-cluster** |  |  |
| 1 | 9.5 (10.2) | 7-15 |
| 2 | 7.5 (7) | 5-9 |
| 3 | 36.5 (37.3) | 26-49 |
| 4 | 20 (20.5) | 14-28 |
| **Three-cluster** |  |  |
| 1 | 17 (17.2) | 14-20 |
| 2 | 36.5 (37.3) | 26-49 |
| 3 | 20 (20.5) | 14-28 |
| **Two-cluster** |  |  |
| 1 | 53.5 (54.5) | 46-63 |
| 2 | 20 (20.5) | 14-28 |
